# Água, saneamento e higiene (WASH): um estudo das escolas públicas rurais de Ensino Fundamental brasileiras

**DOI:** 10.1590/0102-311XPT128025

**Published:** 2026-05-01

**Authors:** Lívia Pita Corrêa, Patrícia Conceição Medeiros, Renato Moreira Hadad, José Irineu Rangel Rigotti, Uende Aparecida Figueiredo Gomes

**Affiliations:** 1 Universidade Federal de Minas Gerais, Belo Horizonte, Brasil.; 2 Pontifícia Universidade Católica de Minas Gerais, Belo Horizonte, Brasil.

**Keywords:** Ensino Fundamental, Saneamento Básico, Escolas, Área Rural, Objetivos do Desenvolvimento Sustentável, Primary Education, Basic Sanitation, Schools, Rural Areas, Sustainable Development Goal, Educación Primaria, Saneamiento Básico, Escuelas, Área Rural, Objetivo de Desarrollo Sostenible

## Abstract

Este estudo investigou a ausência de acesso à água, ao saneamento e à higiene (WASH, acrônimo em inglês) nas escolas públicas rurais de Ensino Fundamental brasileiras, um estudo transversal e comparativo referente aos anos de 2011 e 2023. Por meio de análise descritiva e regressão logística binomial, avaliou-se a distribuição, a evolução temporal e os fatores determinantes dessa ausência, utilizando como principal banco de dados o *Censo Escolar da Educação Básica*. Os resultados revelam desigualdades territoriais persistentes: a Região Norte apresentou as maiores razões de chance de ausência de todos os serviços de WASH em ambos os anos analisados. Escolas localizadas em terras indígenas, com menos de 10 alunos ou com taxas elevadas de abandono e baixos índices de aprovação, apresentaram maiores chances de exclusão sanitária. No nível municipal, destacaram-se como determinantes a elevada proporção de população rural e o baixo Índice de Desenvolvimento Humano Municipal - renda. Apesar de avanços pontuais no acesso à água, retrocessos em outros serviços indicam estagnação ou agravamento das desigualdades. Os resultados reforçam que, passados mais de dez anos, o Brasil rural ainda está distante de cumprir a meta 4.a.1 dos Objetivos de Desenvolvimento Sustentável, que preconiza a universalização do acesso ao WASH no ambiente escolar. Ao revelar os principais entraves institucionais e territoriais, o estudo contribui para subsidiar políticas públicas mais equitativas e sensíveis às especificidades do território rural brasileiro.

## Abstract (en)

This study investigated the lack of access to water, sanitation, and hygiene (WASH) at Brazilian rural public elementary schools through a cross-sectional comparative study of the years 2011 and 2023. The distribution, temporal evolution, and determinant factors of this lack were assessed through descriptive analysis and binomial logistic regression, using the School Census of Basic Education as the main database. The results reveal persistent regional inequalities: the North region had the highest odds ratios for the absence of all WASH services in both years analyzed. Schools located on indigenous lands, with less than 10 students or with high dropout rates and low passing rates, had a greater likelihood of health exclusion. On the municipal level, the high proportion of the rural population and low Municipal Human Development Index - Income stood out as determinants. Despite occasional advances in access to water, setbacks in other services indicate stagnation or the worsening of inequalities. The results reveal that, after more than ten years, rural Brazil is still far from meeting the 4.a.1 target of the Sustainable Development Goals, which stipulates universal access to WASH in the school environment. By revealing the main institutional and regional obstacles, this study contributes to the planning of public policies that are more equitable and sensitive to the specificities of rural areas in Brazil.

## Abstract (es)

Este estudio investigó la falta de acceso al agua, al saneamiento y a la higiene (WASH, por sus siglas en inglés) en las escuelas públicas rurales de educación primaria brasileñas, un estudio transversal y comparativo referente a los años 2011 y 2023. Mediante un análisis descriptivo y una regresión logística binomial, se evaluó la distribución, la evolución temporal y los factores determinantes de esta falta, utilizando como base de datos principal el Censo Escolar de Educación Básica. Los resultados revelan desigualdades territoriales persistentes: la región norte presentó las mayores probabilidades de ausencia de todos los servicios de WASH en ambos años analizados. Las escuelas ubicadas en tierras indígenas, con menos de 10 alumnos o con altas tasas de abandono y bajos índices de aprobación, presentaron mayores probabilidades de exclusión sanitaria. A nivel municipal, se destacaron como factores determinantes la elevada proporción de población rural y el bajo Índice de Desarrollo Humano Municipal-Renta. A pesar de avances puntuales en el acceso al agua, los retrocesos en otros servicios indican un estancamiento o un agravamiento de las desigualdades. Los resultados refuerzan que, tras más de diez años, el Brasil rural aún está lejos de cumplir la meta 4.a.1 de los Objetivos de Desarrollo Sostenible, que aboga por la universalización del acceso al WASH en el ámbito escolar. Al revelar los principales obstáculos institucionales y territoriales, el estudio contribuye a respaldar políticas públicas más equitativas y sensibles a las especificidades del territorio rural brasileño.

## Introdução

O acesso à água, ao saneamento e à higiene (WASH, acrônimo em inglês) nas escolas é fundamental para garantir um ambiente seguro, saudável e inclusivo. No contexto deste estudo, WASH é representado por três dimensões: o abastecimento de água, o saneamento, entendido neste estudo em consonância com a literatura internacional como esgotamento sanitário, e a existência de banheiro, indicadores que refletem diretamente as condições de infraestrutura escolar. A ausência desses serviços, especialmente em áreas rurais, compromete a saúde, o bem-estar e a permanência dos estudantes na escola, além de aprofundar desigualdades estruturais já existentes ^1^ ^,^ ^2^ ^,^ ^3^ ^,^ ^4^. Essa situação evidencia os desafios para o cumprimento da meta 4.a.1 dos Objetivos de Desenvolvimento Sustentável (ODS), que visa assegurar instalações adequadas de WASH em todas as escolas ^5^.

No Brasil, o Ensino Fundamental é uma etapa obrigatória e gratuita nas escolas públicas, cuja faixa etária adequada se estende dos 6 aos 14 anos. Essa fase é subdividida em anos iniciais (1º ao 5º ano) e anos finais (6º ao 9º ano) ^6^. Neste contexto, segundo aponta a literatura ^7^, cerca de 42% das meninas experienciam a menarca entre 8 e 12 anos. Como a maioria delas permanece no ensino fundamental por volta de 3 a 7 anos após a primeira menstruação, evidencia-se a necessidade de instalações adequadas de WASH para promover a equidade de gênero e garantir o bem-estar dessas alunas possibilitando o manejo da menstruação no ambiente escolar ^7^.

Embora estudos sobre WASH no contexto escolar ainda sejam limitados no Brasil, pesquisas apontam deficiências significativas no acesso a esses serviços no país ^8^ ^,^ ^9^ ^,^ ^10^ ^,^ ^11^ ^,^ ^12^ ^,^ ^13^ ^,^ ^14^. A precariedade da infraestrutura sanitária em muitas escolas públicas foi associada a desafios como a falta de água potável, banheiros inadequados e a ausência de condições mínimas para a higiene menstrual, reforçando desigualdades de gênero e impactando principalmente as meninas ^10^. Na América Latina, uma revisão de alcance sobre acesso e práticas de higiene menstrual apontou que a falta de instalações adequadas de água e saneamento se relaciona à redução da frequência escolar, especialmente entre meninas adolescentes ^15^.

Pesquisas em países de renda média e na África Subsaariana mostram que as desigualdades no acesso a WASH escolar são um desafio global. Estudos no Quênia, Uganda, Índia e África do Sul associam a falta de água e saneamento a maiores taxas de absenteísmo e a piores condições de saúde, sobretudo entre meninas, enquanto intervenções em saúde menstrual e melhorias em WASH têm efeitos positivos na frequência e no bem-estar escolar ^16^ ^,^ ^17^ ^,^ ^18^ ^,^ ^19^. Assim, a insuficiência desses serviços permanece como questão estrutural e persistente, refletindo desigualdades socioeconômicas globais.

Em 2021, foi apontado que, em estados como Roraima, Mato Grosso, Acre, Espírito Santo, Pará, Mato Grosso do Sul, Minas Gerais e no Distrito Federal, mais da metade das escolas que atendiam o 9º ano não dispunha de infraestrutura adequada de WASH ^7^. Em 2023, 4% das escolas de ensino fundamental no Brasil não contavam com nenhum serviço de higiene ^20^. Ainda nesse ano, 42% das escolas rurais brasileiras dependiam de fontes de água não seguras ou careciam de acesso a abastecimento, enquanto 9% não dispunham de instalações sanitárias adequadas ou sequer as possuíam ^20^. Em contraste, esses percentuais eram inferiores nas escolas urbanas.

No Brasil, o acesso a WASH na escola tem avançado de forma pontual e pouco integrada entre educação, saúde e infraestrutura. Em 2005, a Fundação Nacional de Saúde (Funasa) lançou o Programa Água na Escola para instalar abastecimento de água e sanitários em escolas rurais, prevendo atender 889 unidades, mas apenas 606 foram contempladas até 2009, indicando limites operacionais e orçamentários ^21^. O Ministério da Educação desenvolveu ações complementares, como o Programa Dinheiro Direto na Escola (PDDE) ^22^ e o Plano de Ações Articuladas (PAR) ^23^, porém a falta de uma política nacional específica e a baixa articulação intersetorial ainda restringem avanços sustentáveis, especialmente em áreas rurais.

Diante desse contexto, este estudo tem como objetivo analisar a ausência de WASH nas escolas públicas rurais de Ensino Fundamental brasileiras, considerando as mudanças ocorridas entre 2011 e 2023. Para isso, busca-se (i) descrever a distribuição da ausência de WASH nas escolas públicas rurais de Ensino Fundamental nesses dois anos; (ii) avaliar a evolução, retrocesso ou estagnação dessa ausência ao longo do tempo; e (iii) identificar as variáveis escolares e municipais determinantes da ausência de WASH nesse período.

Ao investigar esses aspectos, este estudo pretende contribuir para a compreensão das desigualdades no acesso a infraestrutura escolar básica, fornecendo subsídios para políticas públicas que promovam melhores condições sanitárias e educacionais, especialmente em áreas rurais.

## Métodos 

### Área de estudo

Este estudo investiga as condições de infraestrutura sanitária nas escolas públicas rurais de Ensino Fundamental. Em 2011, o Brasil possuía 56.544 escolas nesse nível de ensino, sem contabilizar as escolas que ofertavam Ensino de Jovens e Adultos (EJA), contabilizando um total de 3.008.675 matrículas. Em 2023, esse número caiu para 34.406 escolas púbicas rurais de Ensino Fundamental, com 2.192.038 matrículas, o que representa uma redução de 22.138 escolas publicas rurais de Ensino Fundamental e uma diminuição de 816.637 matrículas ao longo do período analisado ^24^.

### Banco de dados

O *Censo Escolar da Educação Básica*, coordenado pelo Instituto Nacional de Estudos e Pesquisas Educacionais Anísio Teixeira (Inep), foi a principal fonte de dados desta pesquisa transversal e comparativa, que abrange os anos de 2011 e 2023. O *Censo Escolar da Educação Básica* é uma pesquisa estatístico-educacional realizada anualmente, responsável por levantar informações detalhadas sobre a educação básica no Brasil ^25^, amplamente disponibilizadas ao público por meio do site do Inep. Embora seus dados sejam autodeclarados pelos diretores escolares e não passem por auditoria sistemática, o Censo é fundamental para a identificação de padrões e tendências, oferecendo informações abrangentes sobre a infraestrutura escolar, o corpo docente, as matrículas, a jornada escolar, o rendimento e a movimentação dos estudantes, segmentadas por nível, fase e modalidade de ensino ^24^.

Este artigo concentrou-se exclusivamente nas escolas públicas rurais de Ensino Fundamental, selecionando aquelas que permaneceram ativas e vinculadas ao mesmo município entre 2011 e 2023, objetivando evidenciar suas especificidades, analisar as tendências e, eventualmente, subsidiar políticas públicas voltadas à melhoria do acesso a WASH nessas instituições.

A seleção dos anos de 2011 e 2023 para a análise baseou-se em uma análise da consistência e comparabilidade dos microdados do *Censo Escolar da Educação Básica*, bem como em diálogo técnico estabelecido com o Inep. As informações de 2007 a 2010 apresentaram proporções anômalas de escolas sem água, enquanto anos posteriores, como 2017, revelaram inconsistências graves − como variações abruptas na presença de banheiros escolares − reconhecidas pelo Inep como decorrentes de erros no preenchimento do questionário do *Censo Escolar da Educação Básica*. Embora o Inep promova ações de acompanhamento, verificação in loco e validação de dados relacionados aos censos escolares, os dados do *Censo Escolar da Educação Básica* são autodeclaratórios não passam por processos de verificação externa sistemática, o que pode explicar parte dessas inconsistências. Nesse contexto, o ano de 2011 foi selecionado por representar o primeiro com dados considerados mais consistentes, e 2023 por ser o mais recente com informações completas. Já os anos de 2020 e 2021 foram excluídos devido à suspensão das aulas presenciais durante a pandemia de COVID-19.

Os dados municipais foram obtidos a partir dos *Censos Demográficos* de 2010 e 2022, realizados pelo Instituto Brasileiro de Geografia e Estatística (IBGE) ^26^. Também foi consultado o Atlas do Desenvolvimento Humano no Brasil para a obtenção do Índice de Desenvolvimento Humano Municipal (IDHM) - renda ^27^ referentes a 2010, uma vez que, no período da pesquisa, não havia uma base de dados mais recente disponível. 

**Figura 1:**
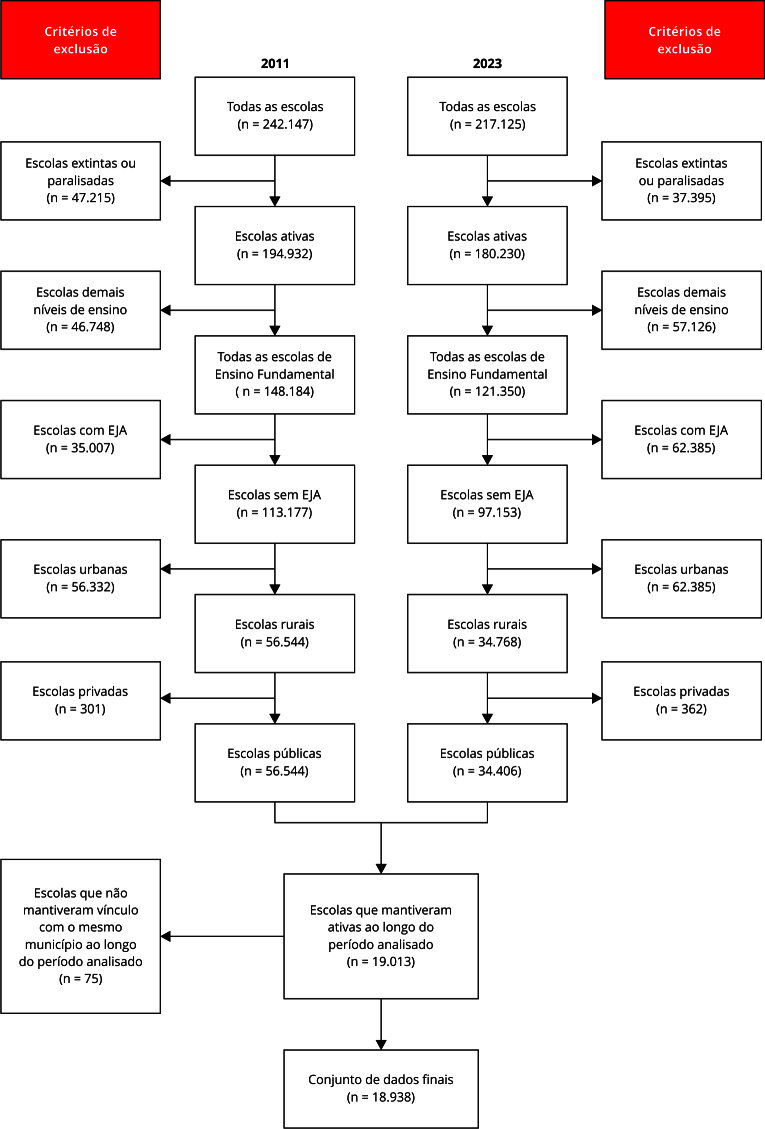
Etapa de organização dos dados do *Censo Escolar da Educação Básica* do Brasil.

O processo de organização do banco de dados do *Censo Escolar da Educação Básica*, que envolveu a seleção de escolas públicas rurais de Ensino Fundamental ativas e vinculadas ao mesmo município entre 2011 e 2023, está detalhado na Figura 1. Essa figura também evidencia uma tendência de paralisação e fechamento escolas públicas rurais de Ensino Fundamental ao longo dos anos.

EJA: Ensino de Jovens e Adultos. Fonte: elaboração própria e adaptado de Instituto Nacional de Estudos e Pesquisas Educacionais Anísio Teixeira ^24^.

### Variáveis analisadas

O Quadro 1 apresenta as variáveis utilizadas nesta pesquisa. As variáveis relacionadas ao nível da escola são, em sua maioria, provenientes do *Censo Escolar da Educação Básica*. Para representar o acesso a WASH, foram consideradas as variáveis abastecimento de água, saneamento e banheiro, uma vez que essas são as únicas disponíveis nesse banco de dados para avaliar, respectivamente, o acesso à água, ao saneamento e à higiene.

**Quadro 1:** Variáveis selecionadas das escolas e dos municípios nos anos de 2011 e 2023.

		VARIÁVEL		CATEGORIA	BASE DE DADOS
Nível da escola	Variáveis sanitárias	Abastecimento de água	Não há abastecimento de água	0 - Não	*Censo Escolar da Educação Básica*
				1 - Sim	
		Banheiro	Existência de banheiro	0 - Não	
				1 - Sim	
		Saneamento	Não há saneamento	0 - Não	
				1 - Sim	
	Variáveis caraterísticas da escola	Identificação da escola		Código da escola	
		Localização diferenciada		0 - A escola não está em área de localização diferenciada	
				1 - Área de assentamento	
				2 - Terra indígena	
				3 - Área onde se localiza comunidade remanescente de quilombos	
				8 - Área onde se localizam povos e comunidades tradicionais *	
		Número de matrículas		Até 10 alunos/Entre 10-20/Entre 20-50/Acima de 50	
		Taxas de rendimento escolar	Aprovação	Até 39%/40%-79%/80% ou mais	Inep
			Reprovação		
			Abandono		
Nível do município	Macrorregião			Centro-oeste/Nordeste/Norte/Sudeste/Sul	*Censo Demográfico*
	Porte populacional do município (habitantes)			Até 10.000/Entre 10.000 e 20.000/Entre 20.000 e 50.000/Entre 50.000 e 100.000/Entre 100.000 e 500.000/Acima de 500.000	
	Percentual de população rural			Até 19%/20%-39%/40%-59%/60%-79%/80%-100%	
	Índice de Desenvolvimento Humano Municipal (IDHM) - renda			Baixo/Médio a Alto **	*Atlas do Desenvolvimento Humano no Brasil* ***

Fonte: elaboração própria, adaptado de Instituto Nacional de Estudos e Pesquisas Educacionais Anísio Teixeira (Inep) ^24^ e AtlasBR ^27^.

* Esta é uma variável nova, presente apenas no ano de 2023, e foi incluída nesta pesquisa por sua importância;

** Categorias de IDHM: baixo (até 0,599), médio a alto (entre 0,6 e 1);

*** *O Atlas do Desenvolvimento Humano no Brasil* é produto da parceria entre o Instituto de Pesquisa Econômica Aplicada e o Programa das Nações Unidas para o Desenvolvimento (PNUD).

É importante destacar que as variáveis do *Censo Escolar da Educação Básica* que tratam do acesso a WASH apresentam codificações específicas. No caso do abastecimento de água, a categoria “Sim” indica que a escola não possui nenhuma fonte de água, ou seja, está totalmente desprovida de acesso à água. Da mesma forma, para saneamento, “Sim” significa que não há nenhum tipo de sistema de saneamento disponível. Já a variável banheiro segue lógica inversa: “Não” indica que a escola não possui banheiro.

### Tratamento estatístico

O tratamento estatístico envolveu análise descritiva da ausência de WASH nas escolas públicas rurais de Ensino Fundamental nos anos de 2011 e 2023, com foco nos serviços de abastecimento de água, saneamento e banheiro. Os resultados foram representados em mapas elaborados no software QGIS, versão 3.28 (https://qgis.org/en/site/), proporcionando visualização espacial das ausências. Para avaliar a evolução, retrocesso ou estagnação dessas condições, foram construídas tabelas cruzadas relacionando as variáveis sanitárias às características escolares e municipais. Essa etapa permitiu um entendimento preliminar das desigualdades existentes entre escolas e municípios. A associação entre as variáveis foi inicialmente verificada por meio do teste qui-quadrado, com nível de 5% de significância, possibilitando identificar relações estatisticamente significativas. Em seguida, aplicou-se a regressão logística binomial para estimar os determinantes da ausência de WASH nos dois anos analisados. Todas as análises estatísticas foram realizadas no software R, versão 4.3.0 (http://www.r-project.org).

## Resultados

### Análise descritiva da ausência de WASH nas escolas públicas rurais de Ensino Fundamental no Brasil

**Figura 2:**
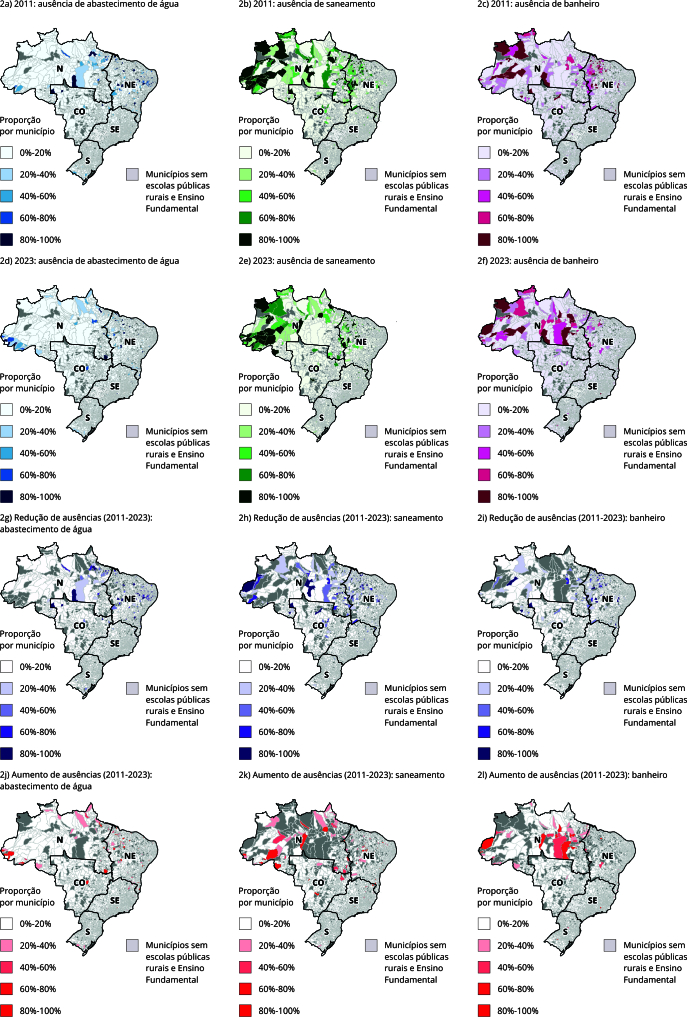
Proporção de escolas públicas rurais de Ensino Fundamental com ausência, redução na ausência e aumento na ausência de abastecimento de água, saneamento e banheiro, por município. Brasil, 2011 e 2023.

CO: Região Centro-oeste; N: Região Norte; NE: Região Nordeste; S: Região Sul; SE: Região Sudeste. Fonte: elaboração própria e adaptado de Instituto Nacional de Estudos e Pesquisas Educacionais Anísio Teixeira ^24^.

A Figura 2 apresenta a proporção de escolas públicas rurais de Ensino Fundamental com ausência de serviços de WASH em 3.424 municípios. Na faixa de 80% a 100%, a macrorregião Nordeste liderou a redução de todas as ausências: abastecimento de água (n = 95, destaque para o Estado de Pernambuco, n = 37), saneamento (n = 21, destaque para o Estado do Maranhão, n = 10) e banheiro (n = 24, destaque para o Estado do Maranhão, n = 12). No mesmo intervalo, o maior aumento da ausência de abastecimento de água foi registrado no Maranhão (n = 9); para o saneamento, destaca-se em Minas Gerais (n = 12); e, no caso dos banheiros, a piora concentrou-se na Região Norte, sobretudo no Pará (n = 6).

### Evolução e tendências da ausência de WASH: relação com variáveis escolares e municipais

A Tabela 1 apresenta o percentual de escolas públicas rurais de Ensino Fundamental com ausência de serviços de WASH, segundo características escolares. Para facilitar a identificação das piores condições, os maiores percentuais foram destacados em tons avermelhados. A classificação das cores foi realizada de forma geral, com o objetivo de evidenciar as variáveis associadas aos maiores percentuais de ausência, considerando cada tipo de ausência sanitária. Em 2011, as maiores ausências de saneamento foram observadas em escolas localizadas em terras indígenas (54,6%) e em escolas com taxa de aprovação de até 39% (56,2%), enquanto a ausência de banheiro chegou a 50% em escolas com taxa de abandono escolar de 80% ou mais. Em 2023, essas desigualdades permaneceram elevadas, com a ausência de saneamento atingindo 69,2% em escolas com taxa de aprovação até 39% e chegando a 75% em escolas com taxa de abandono escolar de 80% ou mais, enquanto a ausência de banheiro aumentou para 83,3% em escolas com taxa de abandono escolar de 80% ou mais.

**Tabela 1:** Percentual das escolas públicas rurais de Ensino Fundamental brasileiras com ausência de abastecimento de água, saneamento e banheiro, segundo as características da escola.

Variáveis/Categoria	Abastecimento de água		Saneamento		Banheiro	
	2011	2023	2011	2023	2011	2023
Localização diferenciada						
0 - A escola não está em área de localização diferenciada	7,9	3,9	9,9	8,2	5,1	4,3
1 - Área de assentamento	8,5	8,8	17	11,5	11,7	13,7
2 - Terra indígena	6,0	13,2	54,6	53,1	41,7	42,8
3 - Área onde se localiza comunidade remanescente de quilombos	14,2	6,6	11,9	4,3	6,6	4,2
8 - Área onde se localizam povos e comunidades tradicionais	NA	0,9	NA	16,3	NA	8,2
Número de matrículas (alunos)						
Até 10	7,7	8,1	17,9	19,3	19,9	13,9
10-20	10,0	6,5	28,0	14,8	16,4	9,9
20-50	10,7	4,8	25,4	11,6	8,2	7,1
Acima de 50	5,1	2,3	3,9	4,1	1,9	2,6
Taxas de aprovação (%)						
Até 39	13,1	9,6	56,2	69,2	22,3	48,1
40-79	10,6	9,3	20,4	25,9	12,3	19,3
80 ou mais	7,3	4,5	10,5	9,7	6,2	6,3
Taxas de reprovação (%)						
Até 39	8,0	4,8	12,4	10,8	7,3	7,2
40-79	9,8	7,0	38,7	35,4	19,5	21,5
80 ou mais	22,2	0,0	33,3	40,0	11,1	0,0
Taxas de abandono (%)						
Até 39	8,0	4,8	12,7	10,8	7,4	7,2
40-79	15,8	23,5	53,9	70,6	39,5	44,1
80 ou mais	0,0	8,3	0,0	75,0	50,0	83,3

Fonte: elaboração própria e adaptado de Instituto Nacional de Estudos e Pesquisas Educacionais Anísio Teixeira ^24^.

NA: dados não disponíveis.

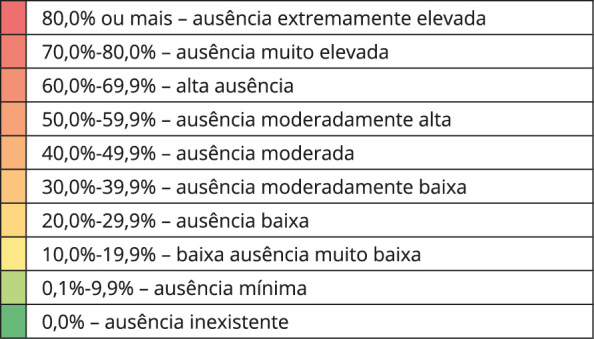

A Tabela 2 revela desigualdades persistentes nas condições de WASH nas escolas públicas rurais de Ensino Fundamental segundo as características municipais, com destaque para a Região Norte, que apresentou as maiores ausências de saneamento e banheiro em 2011 e 2023. Pequenos municípios com alta proporção de população rural e baixo IDHM − renda também registram elevados percentuais de precariedade. Por exemplo, em 2023, a ausência de banheiro nas escolas dos municípios que apresentam mais de 80% de população rural chegou a 27,5%. Como pode ser observado na Tabela 1, os maiores percentuais de ausência foram realçados em tons avermelhados. Esses padrões reforçam a influência das desigualdades regionais e socioeconômicas ao longo do período.

**Tabela 2:** Percentual das escolas públicas rurais de Ensino Fundamental brasileiras com ausência de abastecimento de água, saneamento e banheiro, segundo as características do município.

Categoria	Abastecimento de água		Saneamento		Banheiro	
	2011	2023	2011	2023	2011	2023
Macrorregião						
Centro-oeste	2,3	1,4	5,7	3,4	4,0	2,1
Nordeste	14,6	5,5	11,3	9,6	9,7	4,7
Norte	8,9	10,2	33,1	27,7	15,2	22,2
Sudeste	1,1	1,5	2,1	3,0	0,3	0,4
Sul	1,9	0,5	1,0	0,7	1,1	0,6
Porte populacional do município (habitantes)						
10.000	5,4	4,5	11,9	8,7	7,1	3,4
10.000-20.000	7,6	4,3	11,5	11,0	7,2	6,6
20.000-50.000	9,1	6,7	15,7	12,4	8,7	6,8
50.000-100.000	10,3	3,7	14,0	15,9	8,6	15,6
100.000-500.000	7,2	3,3	8,2	5,7	4,9	5,7
Acima de 500.000	1,7	0,8	2,1	1,2	2,1	6,2
Percentual de população rural						
Até 19	5,0	2,3	2,9	2,7	1,6	1,7
20-39	7,0	4,3	11,3	11,5	6,5	5,5
40-59	9,1	7,2	17,1	16,1	9,4	13,4
60-79	10,4	5,2	15,5	11,1	9,8	4,9
80-100	6,7	5,7	24,5	27,1	18,6	27,5
Índice de Desenvolvimento Humano Municipal − renda						
Baixo	12,8	8,5	24	21,1	13,8	14,3
Médio/Alto	5,1	2,7	6,2	4,9	3,9	3,1

Fonte: elaboração própria, adaptado de Instituto Brasileiro de Geografia e Estatística ^26^ e AtlasBR ^27^.

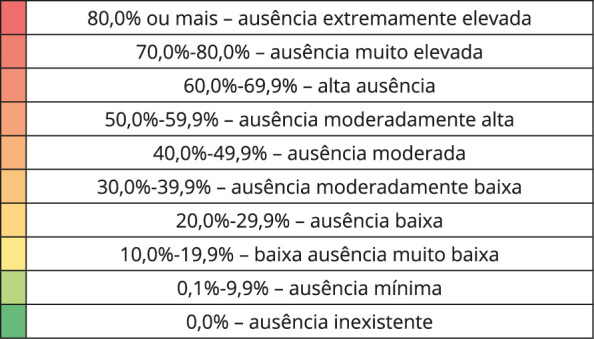

### Fatores determinantes da ausência de WASH nas escolas públicas rurais de Ensino Fundamental

Todas as variáveis não sanitárias, quando avaliadas em pares com as classes das variáveis sanitárias (Quadro 1), por meio dos testes de qui-quadrado, obtiveram significância ao nível de 5%. Dessa forma, todas as classes foram incluídas na análise de regressão logística.

Os resultados indicam significativas desigualdades regionais e contextuais no acesso a serviços de WASH nas escolas púbicas rurais de Ensino Fundamental, tanto em 2011 quanto em 2023. Destacam-se, inicialmente, os resultados por localização regional, considerando a Região Centro-oeste como categoria de referência. Na Região Nordeste, as *odds ratio* (OR) de ausência de abastecimento de água foram elevadas em ambos os anos (OR = 4,84; p < 0,001 em 2011; OR = 3,60; p < 0,001 em 2023). Na Região Norte, a ausência de abastecimento de água também apresentou OR significativas em 2011 (OR = 2,58; p < 0,001) e ainda mais elevadas em 2023 (OR = 6,40; p < 0,001). Além disso, a Região Norte evidenciou OR elevadas de ausência de saneamento (OR = 3,32; p < 0,001 em 2011; OR = 5,48; p < 0,001 em 2023) e para ausência de banheiro em 2023 (OR = 5,17; p < 0,001). Em 2011, porém, a ausência de banheiro não foi estatisticamente significativa (OR = 1,36; p = 0,143). Os achados mostram acirramento das desigualdades na Região Norte, onde as escolas rurais seguem mais expostas à ausência de WASH, contrariando a diretriz de igualdade e não discriminação e revelando desafios persistentes para a universalização do WASH escolar.

Com relação ao território, as escolas públicas rurais de Ensino Fundamental localizadas em terras indígenas apresentaram maiores OR de ausência de saneamento (OR = 5,58; p < 0,001 em 2011; OR = 5,48; p < 0,001 em 2023) e de banheiro (OR = 8,82; p < 0,001 em 2011; OR = 4,55; p < 0,001 em 2023), quando comparadas às demais escolas públicas rurais de Ensino Fundamental, situadas em áreas que não são de localização diferenciada.

No que tange ao porte escolar, as escolas públicas rurais de Ensino Fundamental com até 10 alunos apresentaram maiores razões de chance de ausência de saneamento (OR = 4,97; p < 0,001 em 2011; OR = 2,73; p < 0,001 em 2023), de banheiro (OR = 8,02; p < 0,001 em 2011; OR = 3,41; p < 0,001 em 2023) e, em 2023, também de ausência de abastecimento de água (OR = 2,28; p < 0,001), quando comparadas às escolas com mais de 50 alunos, que servem como categoria de referência. Esses resultados reforçam a associação entre o pequeno porte escolar e a maior vulnerabilidade no acesso aos serviços de WASH.

Em 2011, as características municipais revelaram desigualdades importantes, sendo que municípios com população entre 100 mil e 500 mil habitantes − considerados de porte médio no Brasil − apresentaram maior OR de ausência de abastecimento de água (OR = 4,48; p = 0,004), em comparação com municípios de grande porte, com população superior a 500 mil habitantes.

Por fim, em relação ao desempenho escolar, destacam-se as escolas com taxa de abandono igual ou superior a 80% em 2023, que apresentaram uma alta OR de ausência de banheiro (OR = 7,75; p = 0,045), em comparação àquelas com taxas de abandono entre 40% e 79%. Além disso, escolas com taxa de aprovação de até 39% apresentaram maior OR de ausência de saneamento, em 2011 (OR = 1,79; p = 0,017). Em 2023, porém, a ausência de saneamento não foi estatisticamente significativa (OR = 2,57; p = 0,053).

## Discussão

A análise descritiva revelou desigualdades regionais, precariedade e ausência de acesso a WASH nas escolas públicas rurais de Ensino Fundamental nos dois anos em análise. A Região Norte, especialmente o Estado do Pará, destacou-se negativamente pelo aumento da ausência de banheiros. Tais resultados confirmam a vulnerabilidade de escolas rurais dessas regiões ^11^. De forma preocupante, Minas Gerais, na Região Sudeste − que apresenta a maior cobertura de saneamento domiciliar do país ^28^ registrou o maior número de municípios com ausência de saneamento nas escolas públicas rurais de Ensino Fundamental, revelando desigualdades que persistem mesmo em estados com melhores condições de WASH domiciliar.

A análise das tendências revela desigualdades persistentes no acesso a WASH nas escolas publicas rurais de Ensino Fundamental, associadas a fatores escolares e municipais. Em 2023, as piores condições foram observadas em escolas com baixo desempenho, localizadas em terras indígenas ou com altas taxas de abandono, refletindo padrões mais amplos de exclusão social, como apontado em estudos sobre a desvantagem de domicílios com crianças indígenas no acesso ao WASH ^29^. Escolas situadas em municípios pequenos, com alta proporção de população rural e baixo IDHM − renda, também concentraram as maiores ausências, em consonância com evidências que associam o baixo desenvolvimento municipal à precariedade dos serviços sanitários ^30^. Tais resultados destacam a persistência de desigualdades estruturais que limitam o acesso a direitos essenciais. A Região Norte manteve-se como a mais crítica nos dois anos analisados, reafirmando o papel dos fatores territoriais e socioeconômicos na determinação da precariedade sanitária − tanto nas escolas quanto nos domicílios da região ^28^.

A análise dos fatores determinantes da ausência de serviços de WASH nas escolas públicas rurais de Ensino Fundamental revelou padrões consistentes entre 2011 e 2023, evidenciando desigualdades territoriais e institucionais persistentes. As maiores razões de chance concentraram-se em escolas localizadas na Região Norte, em terras indígenas e com turmas muito pequenas (até 10 alunos), sugerindo que fatores como localização remota e baixo número de matrículas seguem associados a menores condições de investimento em infraestrutura sanitária. Essa relação é coerente com a lógica de financiamento do PDDE, cujos repasses são calculados com base no número de estudantes matriculados ^31^. Estudos indicam que essa fórmula tende a desfavorecer escolas rurais de pequeno porte e dispersas geograficamente, limitando sua capacidade de investimento local ^32^ ^,^ ^33^ ^,^ ^34^.

Em 2023, também se destacaram fatores ligados ao desempenho escolar: escolas com altas taxas de abandono e baixos índices de aprovação apresentaram maior probabilidade de ausência de banheiros, o que está em consonância com a literatura que reconhece a inexistência de instalações sanitárias adequadas − como banheiros privativos e estruturas para higiene menstrual − como barreira à frequência e permanência escolar, especialmente entre meninas, elevando os riscos de estresse psicossocial e infecções urogenitais ^3^ ^,^ ^35^ ^,^ ^36^.

No nível municipal, a ausência de água e banheiros foi mais prevalente em municípios com alta proporção de população rural, refletindo desigualdades estruturais e o frequente desfavorecimento de áreas de difícil acesso geográfico ^37^. Além disso, municípios de médio porte (100 mil a 500 mil habitantes) também apresentaram precariedade, sugerindo que a insuficiência de infraestrutura WASH não se restringe a localidades pequenas, mas envolve questões de gestão, priorização e alocação equitativa de recursos.

## Conclusão

O estudo demonstrou que a ausência de serviços de WASH nas escolas públicas rurais de Ensino Fundamental brasileiras persiste como uma condição desigual. Entre 2011 e 2023, observou-se redução das ausências, sobretudo no abastecimento de água, porém as desigualdades regionais e territoriais mantiveram-se expressivas, com maior concentração na Região Norte e em escolas localizadas em terras indígenas. A análise indicou ainda que escolas de menor porte, com baixos indicadores de aprovação e inseridas em municípios com alta proporção de população rural e baixo IDHM − renda apresentaram maior chance de ausência de serviços de WASH. Esses resultados evidenciam a persistência de fatores estruturais e territoriais que dificultam a universalização do acesso a WASH no ambiente escolar rural.

## Data Availability

As fontes de informação utilizadas no estudo estão indicadas no corpo do artigo.
