# ERRATUM: Água, saneamento e higiene (WASH): um estudo das escolas públicas rurais de Ensino Fundamental brasileiras

**DOI:** 10.1590/0102-311XER128025

**Published:** 2026-06-29

Corrêa LP, Medeiros PC, Hadad RM, Rigotti JIR, Gomes UAF. Água, saneamento e higiene (WASH): um estudo das escolas públicas rurais de Ensino Fundamental brasileiras. Cad Saúde Pública 2026; 42:e00128025.

Onde se lê:

**Tabela 1:** Percentual das escolas públicas rurais de Ensino Fundamental brasileiras com ausência de abastecimento de água, saneamento e banheiro, segundo as características da escola.

Variáveis/Categoria	Abastecimento de água		Saneamento		Banheiro	
	2011	2023	2011	2023	2011	2023
Localização diferenciada						
0 - A escola não está em área de localização diferenciada	7,9	3,9	9,9	8,2	5,1	4,3
1 - Área de assentamento	8,5	8,8	17	11,5	11,7	13,7
2 - Terra indígena	6,0	13,2	54,6	53,1	41,7	42,8
3 - Área onde se localiza comunidade remanescente de quilombos	14,2	6,6	11,9	4,3	6,6	4,2
8 - Área onde se localizam povos e comunidades tradicionais	NA	0,9	NA	16,3	NA	8,2
Número de matrículas (alunos)						
Até 10	7,7	8,1	17,9	19,3	19,9	13,9
10-20	10,0	6,5	28,0	14,8	16,4	9,9
20-50	10,7	4,8	25,4	11,6	8,2	7,1
Acima de 50	5,1	2,3	3,9	4,1	1,9	2,6
Taxas de aprovação (%)						
Até 39	13,1	9,6	56,2	69,2	22,3	48,1
40-79	10,6	9,3	20,4	25,9	12,3	19,3
80 ou mais	7,3	4,5	10,5	9,7	6,2	6,3
Taxas de reprovação (%)						
Até 39	8,0	4,8	12,4	10,8	7,3	7,2
40-79	9,8	7,0	38,7	35,4	19,5	21,5
80 ou mais	22,2	0,0	33,3	40,0	11,1	0,0
Taxas de abandono (%)						
Até 39	8,0	4,8	12,7	10,8	7,4	7,2
40-79	15,8	23,5	53,9	70,6	39,5	44,1
80 ou mais	0,0	8,3	0,0	75,0	50,0	83,3

Fonte: elaboração própria e adaptado de Instituto Nacional de Estudos e Pesquisas Educacionais Anísio Teixeira ^24^.

NA: dados não disponíveis.

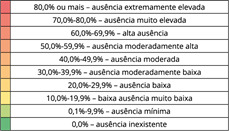

Leia-se:

**Tabela 1:** Percentual das escolas públicas rurais de Ensino Fundamental brasileiras com ausência de abastecimento de água, saneamento e banheiro, segundo as características da escola.

Variáveis/Categoria	Abastecimento de água		Saneamento		Banheiro	
	2011	2023	2011	2023	2011	2023
Localização diferenciada						
0 - A escola não está em área de localização diferenciada	7,9	3,9	9,9	8,2	5,1	4,3
1 - Área de assentamento	8,5	8,8	17	11,5	11,7	13,7
2 - Terra indígena	6,0	13,2	54,6	53,1	41,7	42,8
3 - Área onde se localiza comunidade remanescente de quilombos	14,2	6,6	11,9	4,3	6,6	4,2
8 - Área onde se localizam povos e comunidades tradicionais	NA	0,9	NA	16,3	NA	8,2
Número de matrículas (alunos)						
Até 10	7,7	8,1	17,9	19,3	19,9	13,9
10-20	10,0	6,5	28,0	14,8	16,4	9,9
20-50	10,7	4,8	25,4	11,6	8,2	7,1
Acima de 50	5,1	2,3	3,9	4,1	1,9	2,6
Taxas de aprovação (%)						
Até 39	13,1	9,6	56,2	69,2	22,3	48,1
40-79	10,6	9,3	20,4	25,9	12,3	19,3
80 ou mais	7,3	4,5	10,5	9,7	6,2	6,3
Taxas de reprovação (%)						
Até 39	8,0	4,8	12,4	10,8	7,3	7,2
40-79	9,8	7,0	38,7	35,4	19,5	21,5
80 ou mais	22,2	0,0	33,3	40,0	11,1	0,0
Taxas de abandono (%)						
Até 39	8,0	4,8	12,7	10,8	7,4	7,2
40-79	15,8	23,5	53,9	70,6	39,5	44,1
80 ou mais	0,0	8,3	0,0	75,0	50,0	83,3

Fonte: elaboração própria e adaptado de Instituto Nacional de Estudos e Pesquisas Educacionais Anísio Teixeira ^24^.

NA: dados não disponíveis.

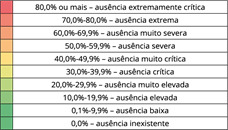

Onde se lê:

**Tabela 2:** Percentual das escolas públicas rurais de Ensino Fundamental brasileiras com ausência de abastecimento de água, saneamento e banheiro, segundo as características do município.

Categoria	Abastecimento de água		Saneamento		Banheiro	
	2011	2023	2011	2023	2011	2023
Macrorregião						
Centro-oeste	2,3	1,4	5,7	3,4	4,0	2,1
Nordeste	14,6	5,5	11,3	9,6	9,7	4,7
Norte	8,9	10,2	33,1	27,7	15,2	22,2
Sudeste	1,1	1,5	2,1	3,0	0,3	0,4
Sul	1,9	0,5	1,0	0,7	1,1	0,6
Porte populacional do município (habitantes)						
10.000	5,4	4,5	11,9	8,7	7,1	3,4
10.000-20.000	7,6	4,3	11,5	11,0	7,2	6,6
20.000-50.000	9,1	6,7	15,7	12,4	8,7	6,8
50.000-100.000	10,3	3,7	14,0	15,9	8,6	15,6
100.000-500.000	7,2	3,3	8,2	5,7	4,9	5,7
Acima de 500.000	1,7	0,8	2,1	1,2	2,1	6,2
Percentual de população rural						
Até 19	5,0	2,3	2,9	2,7	1,6	1,7
20-39	7,0	4,3	11,3	11,5	6,5	5,5
40-59	9,1	7,2	17,1	16,1	9,4	13,4
60-79	10,4	5,2	15,5	11,1	9,8	4,9
80-100	6,7	5,7	24,5	27,1	18,6	27,5
Índice de Desenvolvimento Humano Municipal − renda						
Baixo	12,8	8,5	24	21,1	13,8	14,3
Médio/Alto	5,1	2,7	6,2	4,9	3,9	3,1

Fonte: elaboração própria, adaptado de Instituto Brasileiro de Geografia e Estatística ^26^ e AtlasBR ^27^.

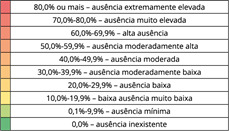

Leia-se:

**Tabela 2:** Percentual das escolas públicas rurais de Ensino Fundamental brasileiras com ausência de abastecimento de água, saneamento e banheiro, segundo as características do município.

Categoria	Abastecimento de água		Saneamento		Banheiro	
	2011	2023	2011	2023	2011	2023
Macrorregião						
Centro-oeste	2,3	1,4	5,7	3,4	4,0	2,1
Nordeste	14,6	5,5	11,3	9,6	9,7	4,7
Norte	8,9	10,2	33,1	27,7	15,2	22,2
Sudeste	1,1	1,5	2,1	3,0	0,3	0,4
Sul	1,9	0,5	1,0	0,7	1,1	0,6
Porte populacional do município (habitantes)						
10.000	5,4	4,5	11,9	8,7	7,1	3,4
10.000-20.000	7,6	4,3	11,5	11,0	7,2	6,6
20.000-50.000	9,1	6,7	15,7	12,4	8,7	6,8
50.000-100.000	10,3	3,7	14,0	15,9	8,6	15,6
100.000-500.000	7,2	3,3	8,2	5,7	4,9	5,7
Acima de 500.000	1,7	0,8	2,1	1,2	2,1	6,2
Percentual de população rural						
Até 19	5,0	2,3	2,9	2,7	1,6	1,7
20-39	7,0	4,3	11,3	11,5	6,5	5,5
40-59	9,1	7,2	17,1	16,1	9,4	13,4
60-79	10,4	5,2	15,5	11,1	9,8	4,9
80-100	6,7	5,7	24,5	27,1	18,6	27,5
Índice de Desenvolvimento Humano Municipal − renda						
Baixo	12,8	8,5	24	21,1	13,8	14,3
Médio/Alto	5,1	2,7	6,2	4,9	3,9	3,1

Fonte: elaboração própria, adaptado de Instituto Brasileiro de Geografia e Estatística ^26^ e AtlasBR ^27^.

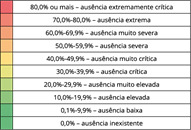

Responsáveis pela aprovação:

Editora-chefe Marilia Sá Carvalho

Editora-chefe Luciana Dias de Lima

Editora-chefe Luciana Correia Alves

